# Building on a Solid Baseline: Anticipatory Biases in Attention

**DOI:** 10.1016/j.tins.2018.01.005

**Published:** 2018-03

**Authors:** Anna C. Nobre, John T. Serences

**Affiliations:** 1 Department of Experimental Psychology Department, University of Oxford, Oxford, UK; 2 Oxford Centre for Human Brain Activity, Department of Psychiatry, Wellcome Centre for Integrative Neuroimaging, University of Oxford, Oxford, UK; 3 Psychology Department, University of California San Diego, La Jolla, CA, USA; 4 Neurosciences Graduate Program, University of California San Diego, La Jolla, CA, USA; 5 Kavli Institute for Brain and Mind, University of California, San Diego, La Jolla, CA 92093, USA

**Keywords:** expectation, anticipatory bias, multivariate pattern analysis, encoding models, visual cortex, fMRI

## Abstract

A brain-imaging paper by Kastner and colleagues in 1999 was the first to demonstrate that merely focusing attention at a spatial location changed the baseline activity level in various regions of human visual cortex even before any stimuli appeared. The study provided a touchstone for investigating cognitive–sensory interactions and understanding the proactive endogenous signals that shape perception.

Our perception derives from the interaction between incoming sensory stimulation and endogenous factors linked to task goals, expectations, and memories. Selective attention comprises the functions that prioritize and select relevant information from the incoming sensory stream based on these endogenous signals and, thus, is an essential building block of cognition. In 1999, *Neuron* published a brain-imaging study by Kastner and her colleagues [1] that significantly advanced our understanding of selective attention in the human brain. In their task, participants viewed colored stimulus patterns appearing sequentially or simultaneously in four locations of the upper right-hand quadrant, and were instructed to detect a prespecified stimulus pattern (target) appearing at a given, fixed location. Similar to previous and contemporaneous studies, results highlighted the involvement of dorsal parietal and premotor–prefrontal areas in controlling spatial attention, and revealed modulation throughout multiple visual areas, including the primary visual cortex. In addition, the study made two important novel contributions. By framing the study within theoretical and methodological approaches developed using nonhuman primate models, the results supported two central tenets of the influential biased-competition model of selective attention [2].

The first tenet of the biased-competition model of attention [2] is the existence of an anticipatory signal that biases the analysis of incoming sensory stimuli. This anticipatory signal is based on goal-related stimulus templates and, in the context of visual processing, facilitates processing of the visual features and spatial locations of task-relevant items. Kastner and colleagues [1] were the first to observe a putative anticipatory biasing signal in the human brain. Even before any stimulus was presented in the trials, significant tonic elevation of brain activity occurred in visual areas responsive to the task-relevant spatial location, as well as in dorsal frontal and parietal areas implicated in controlling spatial attention [3]. This ‘baseline shift’ was compatible with spatially selective preactivation in early visual areas to facilitate subsequent processing of the relevant target stimulus. Notably, and somewhat curiously, the observed baseline shift was pronounced, whereas in previous single-unit recordings in animal studies, prestimulus modulations were often modest or even absent [4]. The reasons for discrepancies in the nature and magnitude of effects in imaging versus single-unit studies are not entirely settled [5]. Nevertheless, the finding of Kastner and colleagues provided persuasive evidence for preparatory attention signals and, thus, represents one of the first important novel contributions from human FMRI studies.

The second, related tenet of the biased-competition model is that attentional modulation is primarily directed at resolving competition among visual stimuli. Accordingly, Kastner and colleagues observed greater attentional modulation when stimuli appeared simultaneously and, thus, competed for neural processing, than when they appeared sequentially and competition was (presumably) minimal. The stimulus-related hemodynamic response in multiple visual areas was significantly larger when the stimuli were attended compared with when they were passively viewed in a control condition. This difference between attended and unattended conditions was accentuated when stimuli competed for visual processing. Although it remains puzzling to understand the mapping between modulations at the single-neuron level and those observed at the regional level using hemodynamic signals, the study suggested that population-imaging measures preserved important functional properties seen at the cellular level.

The findings have stood the test of time, and provided an anchor point for the refinement of our knowledge and understanding of the neural basis of attention. The dorsal frontoparietal network implicated in the control of attention [3] is investigated with increasing granularity, subcortical areas are recognized to integrate network activity, and brain-stimulation studies probe the causal influence of its constituent functional areas on visual processing 6, 7, 8. The plurality of modulatory sites is ever more striking as the sensitivity of imaging methods increases 7, 9. It is intuitive to propose that sensory modulation starts in brain areas processing stimulus attributes that are relevant to task goals and that differentiate target from competing distractor stimuli. However, in reality, tracking the evolution of neural modulation within the nodes of the rich and highly interconnected visual network remains challenging. This will be a fruitful area for investigation with methods that sample multiple sites simultaneously with high spatial and temporal resolution (e.g., simultaneous intracranial recordings or electrocorticography).

Human MRI methods improve relentlessly. In addition to ever-increasing improvements in hardware and imaging sequences, research over the past decade has revolutionized analytical methods, enabling researchers to investigate the informational content within brain areas and networks, and to relate it to behavioral performance at the level of single trials. Multivariate methods were developed to compare the pattern of small variations in the fMRI signal within a population of imaging units (voxels). Using multivariate pattern analysis, attention-related anticipatory biasing signals were shown to share informational content with the anticipated target stimulus within visual areas [10]. Going further, the use of multivariate methods to derive encoding models based on tuning functions of voxels [11] has enabled more precise investigations into the nature of anticipatory and modulatory signals. These methods have also been adapted for human electrophysiology with electro- and magnetoencephalography (EEG and MEG), which have the necessary temporal resolution to chart the temporal dynamics of anticipatory and modulatory signals as they unfold [12].

Findings based on these novel analytical methods have led us away from the long-held notion of attention-related anticipatory preparation carried by a static sustained signal that preactivates ensembles of neurons based on receptive fields matching the spatial locations or features of goal-related stimulus templates. Instead, anticipatory control has revealed itself to be more flexible, adaptive, and dynamic than had been previously assumed. For example, in a functional MRI task requiring participants to decide whether the orientation or contrast of two peripheral gratings matched, analysis using an encoding model revealed that foreknowledge about stimulus orientation could increase activity in neuronal populations coding nontarget orientations [13]. When off-target orientations were particularly useful to guide performance, activity in their population receptive fields were elevated and correlated with behavioral performance. Thus, rather than simply preactivating neuronal populations receptive to target-related templates, attention proactively and selectively prepares neuronal populations that are most informative. Complementing MRI studies, human neurophysiology has revealed the dynamic nature and time course of attention-related biases. MEG recordings taken when individuals matched incoming visual orientation stimuli against a mental template revealed reliable dynamic trajectories of brain activity patterns carrying template-related content [14]. Rather than being sustained, the decoding of the template content ebbed and flowed with the temporal rhythm of stimulus presentation, suggesting the possibility of latent codes that become ‘energized’ by temporal expectations and incoming sensory stimulation [15].

The recent discoveries outlined above have prompted researchers to reconsider the idea of tonic delay activity. Under the hood of seemingly sustained signals over an average of trials lie many interesting and non-mutually exclusive possibilities of short-lived bursts of processing, dynamic sequences of activations, reverberating network states, and latent traces of stimulus information left behind by short-term synaptic plasticity. Whatever form these signals take, they are not merely passive reproductions of memory templates, but rather active prospective constructs to facilitate goal-based adaptive behavior.
